# Experiences with waterjet hydrosurgery system in wound debridement

**DOI:** 10.1186/1749-7922-2-10

**Published:** 2007-05-02

**Authors:** Raffi Gurunluoglu

**Affiliations:** 1 Plastic and Reconstructive Surgery, Denver Health Medical Center, University of Colorado Health Sciences, Denver, Colorado, USA

## Abstract

**Background:**

Recently, a new device, the Versajet™, involving "Hydrosurgery Technology" which combines lavage and sharp debridement instrumentation has been described for soft tissue debridement.

**Methods:**

The Versajet™ Hydrosurgery System utilizes a reusable power console with foot pedal activation, disposable handpiece and tubing assembly in conjunction with sterile saline and standard waste receptacle. The purpose of this paper is to report our experiences with this instrument in debridement of a variety of wounds prior to final reconstructive surgery. Technical details and pitfalls are discussed to facilitate clinical use.

**Results:**

Efficient, safe and fast debridement was achieved in all patients using the hydrosurgery system. The actual time the hydrosurgery system was used for debridement averaged as 15.5 minutes. In ten patients, an adequately debrided wound bed was achieved with a single operative procedure, in four patients; two stages were required prior to reconstructive surgery. In one patient with recurrent sacral-iscial pressure sore, two debridements were carried out followed by long term vacuum assisted closure. The postoperative course was uneventful in all patients, but in three with a minor breakdown of the skin graft, which eventually healed with no surgical intervention.

**Conclusion:**

As a result of our clinical experience, the Versajet™ enables surgeon to precisely target damaged and necrotic tissue and spare viable tissue. This modality may be a useful alternative tool for soft tissue debridement in certain cases. However, further studies are required to investigate its cost-effectiveness in wound management.

## Background

Surgical debridement of necrotic tissue is an essential part of wound care prior to any reconstructive options. Sharp techniques utilized for this purpose have been the mainstay and are commonly used in combination with pulsed lavage and/or irrigation.

Recently, a new device, the Versajet™ Hydrosurgery system, has been described for debridement. The Versajet™ hydrosurgery system utilizes high fluid technology for wound debridement. To date, few papers evaluating its role have been published in the literature [1-4]. The purpose of this paper is to report our experiences with this instrument in debridement of a variety of wounds prior to final reconstructive surgery. Technical details and pitfalls are discussed to facilitate clinical use.

## Patients and methods

### Hydrosurgery system

**Figure 1:**
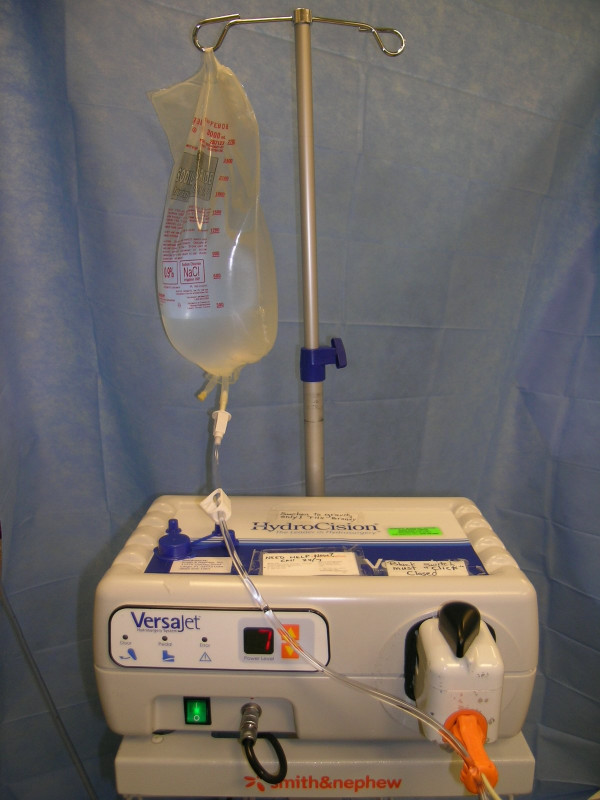
The Versajet™ hydrosurgery console. The system utilizes a reusable power console with foot pedal activation, disposable handpiece and tubing assembly in conjunction with sterile saline.

**Figure 2:**
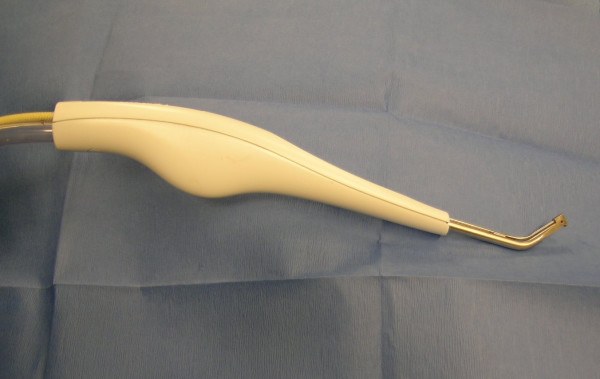
The disposable handpiece (15°/14 mm) used for debridement.

**Figure 3:**
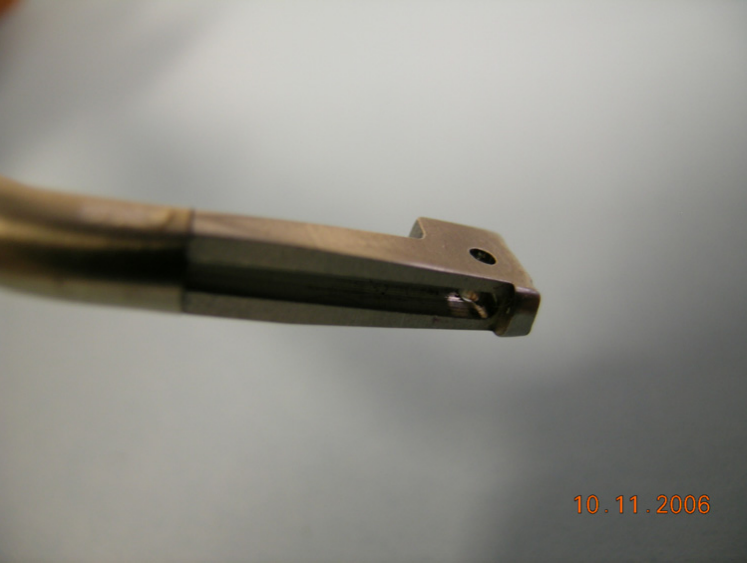
Close-up view of the handpiece.

**Figure 4:**
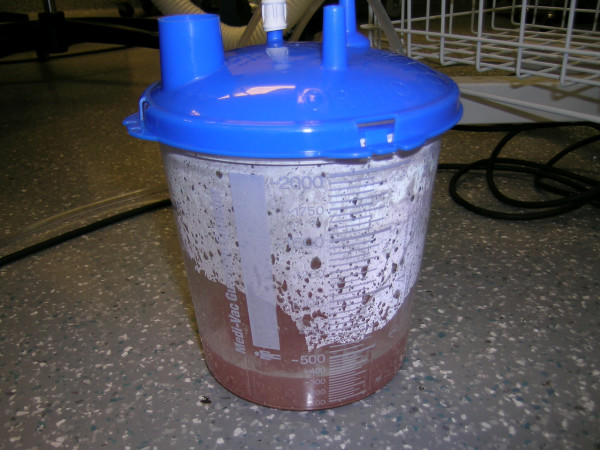
The receptacle in which the debri and aspiration fluid are collected.

The Versajet™ hydrosurgery system was provided by Smith & Nephew, Inc. Colorado. The US Food and Drug Administration (FDA) approved this new surgical instrument that utilizes a high powered parallel waterjet for wound debridement. The system consists of a reusable power console with foot pedal activation, disposable hand piece (15°/14 mm, 45°/14 mm, 45°/8 mm) and tubing assembly in conjunction with sterile saline and standard waste receptacle for maximized effectiveness (Fig. 1, 2, 3, 4).

The hydrosurgery system projects a high-velocity waterjet across the operating window into an evacuation collector thereby creating a localized vacuum. The suction permits the surgeon to hold and cut targeted tissue while aspirating debris from the site. The cutting and aspiration effects can be controlled by adjusting console power settings (10: highest, 1: lowest), handpiece orientation, and handpiece pressure

The hydrosurgery system was used for debridement of fifteen wounds in fifteen patients: 2 venous ulcers (distal leg extending to the foot dorsum, and the middle third of the leg), 1 sacral-iscial pressure sore, 1 burn wound to hand, 1 traumatic scalp wound, 2 traumatic thigh and/or popliteal fossa wounds, 2 traumatic leg wounds, 1 thigh wound following necrotizing fasciitis (Shooter's abscess), 1 leg wound following necrotizing fasciitis (Diabetic), 1 traumatic elbow wound, 1 traumatic foot wound, 1 traumatic arm wound, and 1 above knee amputation stump wound (Table 1). For all cases, a standard handpiece with a 45 degree angled tip and a 14 mm working window was employed. Four cases that underwent debridement with the hydrosurgery system are presented in the following section.

**Table 1:** The table displays a summary of wound characteristics in sixteen patients, for which the hydrosurgery system was used for debridement, including the final reconstructive surgery.

**Patients and age (yrs)**	**Wound Site**	**Wound Size (cm)**	**Etiology**	**Power setting**	**Reconstruction**
P1:44 (m)	Lt Leg	10 × 15	Venous stasis	3	STSG
P2:42 (f)	Lt hand	8 × 8	Burn	3 and 2	Thick STSG
P3:43 (f)	Lt temporo-parietal scalp	4 × 6	Traumatic	5	Rotational scalp flap
P4:50 (f)	Lt elbow	5 × 7	Traumatic	5	Lateral arm island flap
P5:35 (f)	Rt distal thigh and poplitea,	15 × 25	Traumatic	6 and 5	Medial Gastrocnemius muscle flap and STSG
P6:48 (m)	Sacral-ischial	10 × 10	Pressure sore	9 and 8	V.A.C.
P7:44 (f)	Lt distal leg	6 × 10	Traumatic	6	STSG
P8: 55 (m)	Rt distal leg and foot dorsum	10 × 25	Venous stasis	4 and 3	STSG
P9:30 (m)	Lt thigh	10 × 30	Necrotizing fasciitis after shooter's abscess	5	STSG
P 10: 26 (f)	Lt medial arm	6 × 9	Traumatic	3	STSG
P 11: 42 (m)	Lt leg	5 × 5	Traumatic	3	STSG
P 12: 53 (m)	Rt thigh	30 × 30	Traumatic	5 and 4	STSG
P 13: 54 (m)	Lt above knee amputation stump	10 × 15	Traumatic	4	Free Latissimus Dorsi musle flap
P 14: 18 (m)	Lt foot dorsum	3 × 9	Traumatic	4	STSG
P 15: 42 (m)	Lt leg	10 × 30	Necrotizing fasciitis (Diabetic)	6	STSG

(P: Patient, m: male, f: female, Lt: Left, Rt: Right, STSG: Split-thickness skin grafting, V.A.C.: Vacuum assisted closure).

#### Patient 1

**Figure 5:**
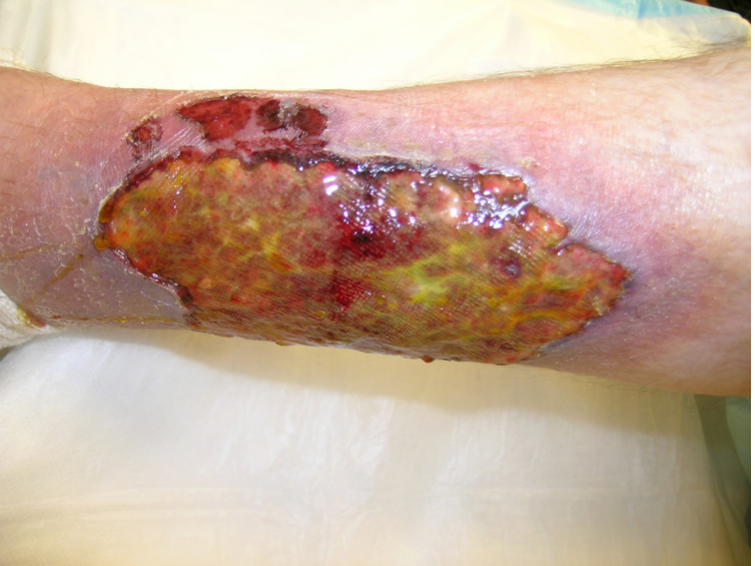
Chronic non-healing wound on the left leg.

**Figure 6:**
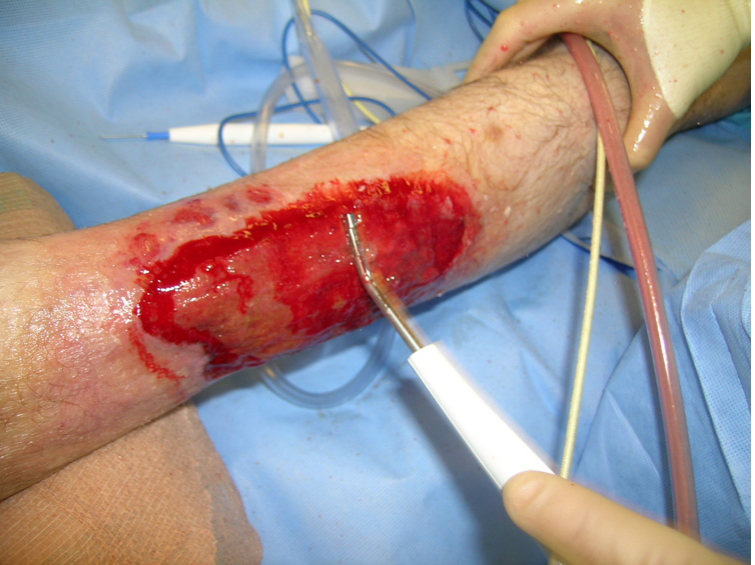
The wound after debridement with the Versajet™ hydrosurgery system.

**Figure 7:**
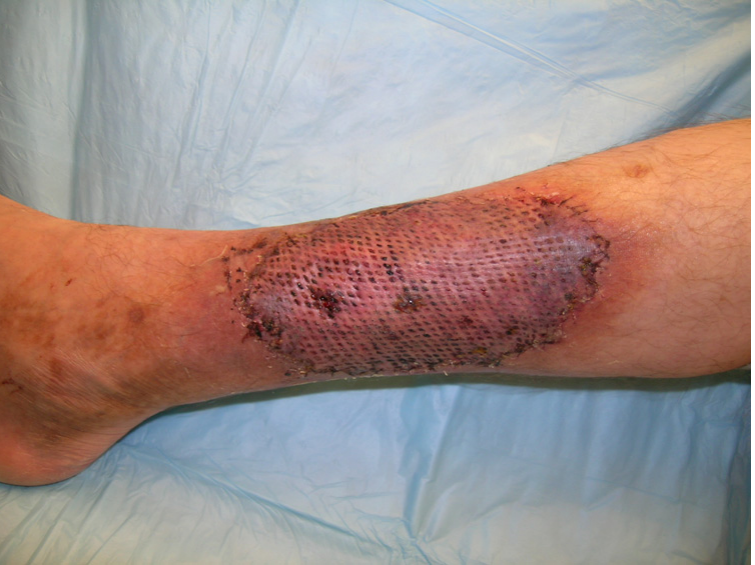
Early postoperative picture showing the full take of the skin graft.

This was a 44-year-old male patient who presented to the Plastic Surgery outpatient clinic with a chronic non-healing wound on the left leg (10 × 15 cm) due to venous stasis. The wound had been present for three months and exhibited positive methicillin resistant staphylococcus aureus (MRSA) cultures on two occasions. The MRSA was treated with parenteral as well as oral antibiotics. Past medical history was significant for moderate rheumatoid arthritis for which the patient was taking 5 mg prednisone daily. The patient's wound was initially treated using wet to dry dressings for five days. Next, a single debridement with the Versajet™ hydrosurgery system (Power setting: 5) was performed followed by immediate split-thickness skin grafting. Early postoperative course was uneventful with full graft take (Fig. 5, 6, 7).

#### Patient 2

**Figure 8:**
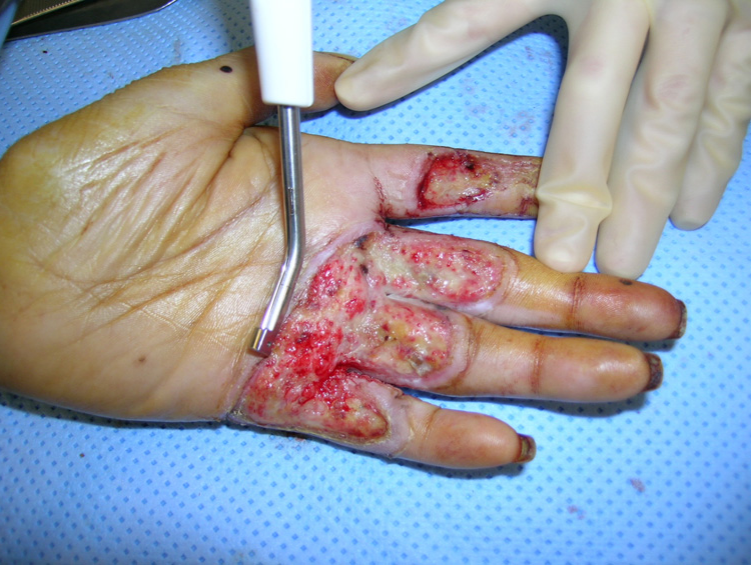
Debridement of the burn wound with the Versajet™ hydrosurgery system.

**Figure 9:**
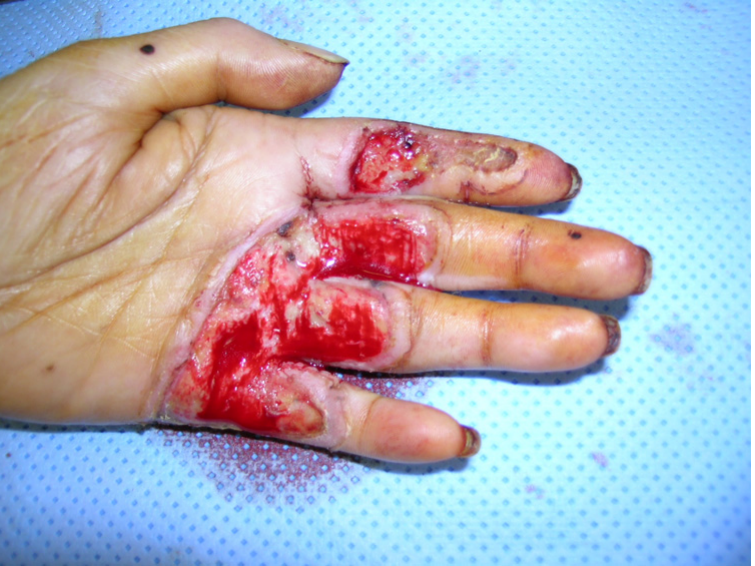
The healthy wound bed after the Versajet™ was used.

**Figure 10:**
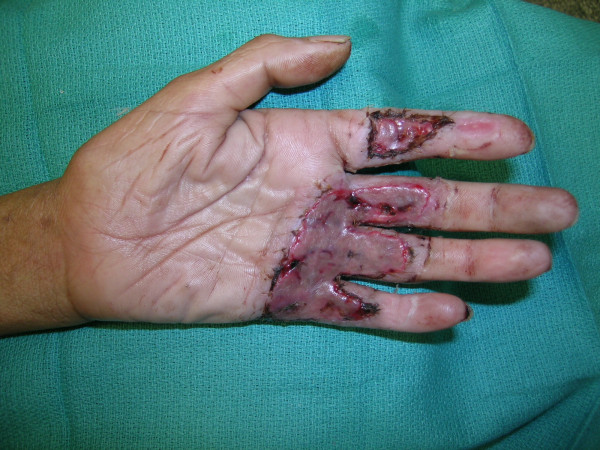
One week after thick split-thickness skin grafting with full take.

This was a 42-year-old female patient who had a third degree burn injury to the left hand following a motor cycle accident. The injury affected the volar and proximal aspect of index, middle, ringer, little finger and distal palmar regions. An initial escharectomy was followed by two debridements using the hydrosurgery system (Power setting: 2–3). Subsequently, a thick partial thickness skin grafting was performed with full take (Fig. 8, 9, 10). Physical therapy was started within ten days after skin grafting with no disability or contracture formation at 3 month-follow-up.

#### Patient 4

**Figure 11:**
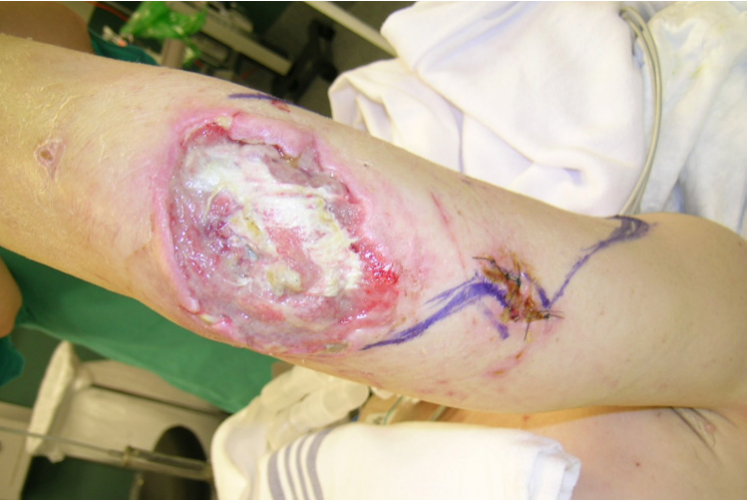
Traumatic right elbow wound with exposed olecranon.

**Figure 12:**
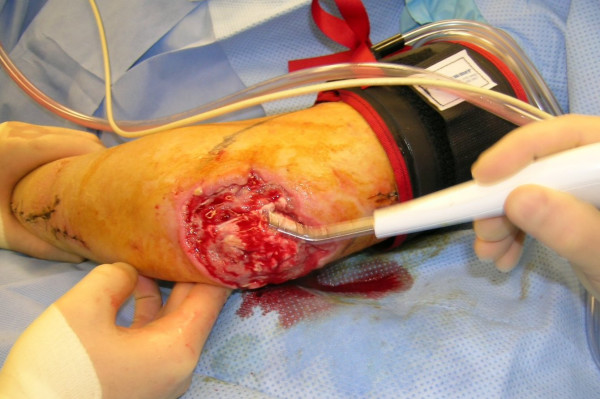
Debridement of the elbow wound with hydrosurgery system.

**Figure 13:**
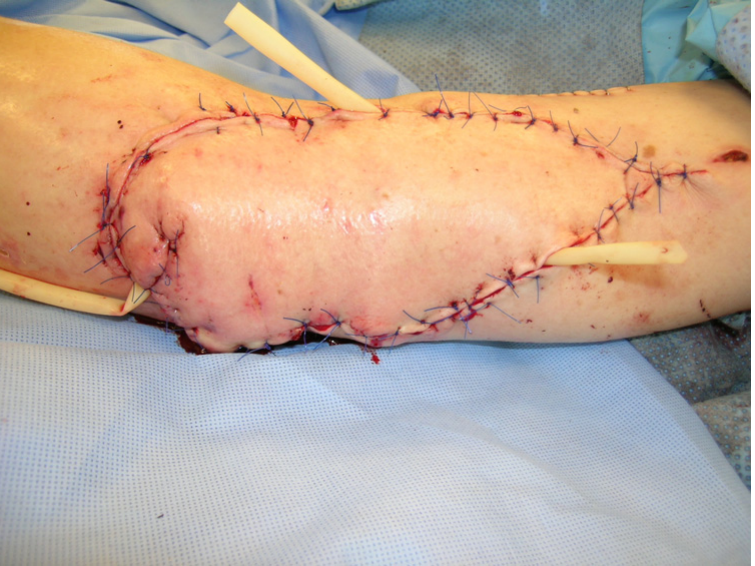
Immediate reconstruction with lateral arm island flap following debridement.

This was a 50-year-old female patient with a traumatic left elbow wound due to a motor vehicle accident. The soft tissue defect was 5 × 7 cm in size with exposed olecranon. Because of her additional abdominal injury, a wound vacuum assisted closure (Vacuum-assisted closure (V.A.C), KCI, San Antonio, TX) was placed. Within three days after VAC treatment, debridement with the hydrosurgery system (Power setting: 5) was performed followed by immediate reconstruction with lateral arm island flap (Fig. 11, 12, 13). Physical therapy was initiated at one week after the surgery. The patient regained elbow functions in 2 months.

#### Patient 5

**Figure 14:**
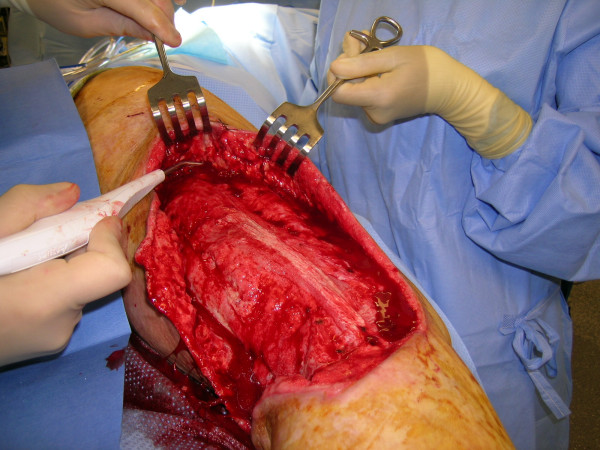
Debridement of the underneath of the avulsion skin flaps with the Versajet™ handpiece, in addition to wound bed preparation.

**Figure 15:**
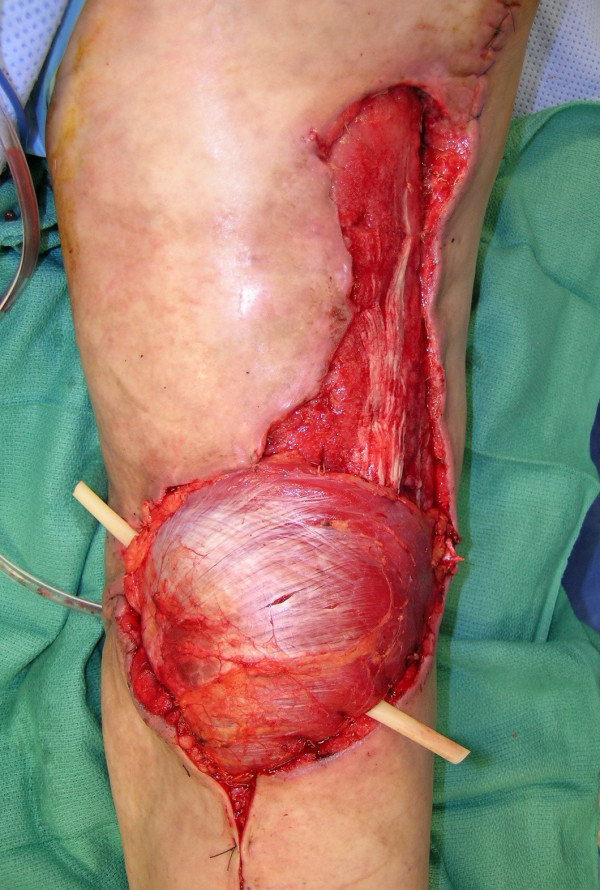
Reconstruction of the popliteal defect and lateral aspect of the knee with medial gastrocnemius muscle flap.

**Figure 16:**
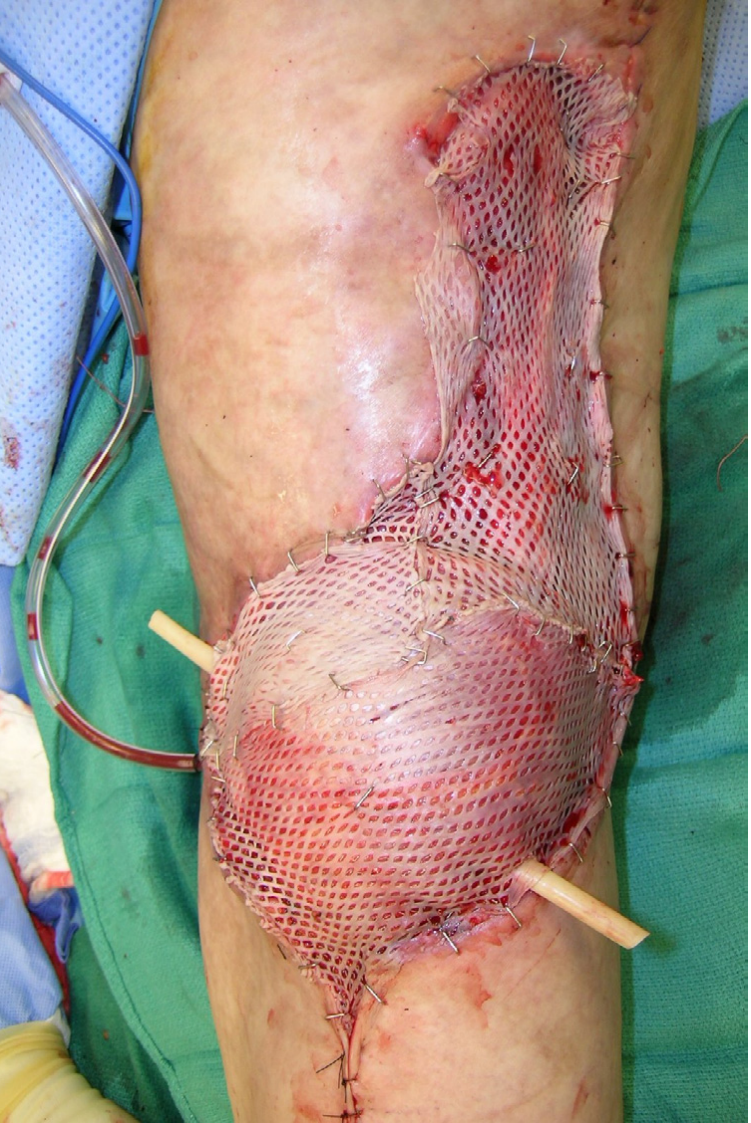
Reconstruction was completed with split-thickness skin grafting.

This was a 30-year-old female patient with a traumatic degloving injury (15 × 25 cm) involving distal thigh and popliteal fossa extending to the lateral aspect of the knee on the right side. The mechanism of injury was a motor vehicle collision. The Versajet™ hydrosurgery system was used twice for wound debridement. The wound edges had 6–7 cm of dissection medially, laterally, and superiorly. The Versajet™ was also used for debridement under the dissected skin edges (Power setting: 6). The underneath of the avulsion skin flaps were tacked down to the wound bed to obliterate the dead space. The distal thigh wound was skin grafted, and the popliteal wound and lateral aspect of the knee were reconstructed with medial gastrocnemius muscle flap covered with split-thickness skin graft (Fig. 14, 15, 16). Minor wounds healed with no complications at three weeks.

## Results

The Versajet™ hydrosurgery system was used for soft tissue debridement in fifteen patients with a variety of wounds. Demographics, wound characteristics, and type of the reconstructive surgery are displayed in Table 1. The patients' age ranged from 18 to 55 years, with an average of 39.1 years. All patients had debridements performed under general anesthesia in an operating room. In ten patients, an adequately debrided wound bed was achieved with a single operative procedure. In four patients, two steps were required before the final reconstructive surgery. In one patient with long-standing recurrent sacral-iscial pressure and poor general status, after dry eschar was removed, two debridements were carried out followed by long term V.A.C. application. No postoperative infection was noted in any of the patients. In three patients, minor breakdown of the skin graft was observed; however, they healed with no surgical intervention. The estimated average time per hydrosurgical procedures was 15.5 minutes. For the cases presented, a standard handpiece with a 45 degree angled tip and a 14 mm working window was employed. The lowest power setting used for debridement was 2–3 (hand burn case: patient 2), and the highest power setting was 8–9 for debridement of a deep pressure sore (patient 6). No safety issues such as sharp injuries or splash contamination were reported during the use of the Versajet™.

## Discussion

Few articles have been published about the hydrosurgery system. The high-powered waterjet is a unique device compared to the pulse irrigator, which is a low-energy waterjet. The high-powered parallel waterjet device has the ability to focus a high-powered stream of water into a high-energy cutting implement. Water dissection works by the Venturi effect. A jet of saline, propelled by a power console, travels across the operating window of a hand-held piece and then into a suction collector. This system of pressurized saline functions like a knife. The saline beam is aimed parallel to the wound so that the cutting mechanism is a highly controlled form of tangential excision [4].

We experienced many advantages utilizing the hydrosurgery system in fifteen patients. This single device technique combines lavage and sharp debridement instrumentation with single-handed operation due to holding and treating with one device. The device provided the control to hold targeted tissue during irrigation and excision, and importantly, the handpiece provided the ability to perform simultaneous debridement as well as removal of debris by aspiration. This helped keep the operative field cleaner and drier compared to conventional lavage techniques.

The highly selective form of tangential excision enables surgeon to precisely target damaged and necrotic tissue and spare viable tissue. Furthermore, the hydrosurgery system offers multiple power settings for controlled excision around delicate tissues.

The Versajet™ hydrosurgery system enables the surgeon to accurately control the cutting, debriding and aspiration effects by adjusting the console power settings from 1 to 10 as well as by angulating the hand piece. Increasing the power setting decreases the duration of debridement, whereas decreasing the power setting increases the duration of debridement. Alternating pressure of the handpiece can further modulate its use and effect on the wound surface. It would be safer if one starts with a lower setting and makes appropriate adjustments based on the individual wound being excised.

Preservation of nerves, vessels, and tendons is of utmost importance when performing debridement in the hand (as in patient 2), and surgical precision and control of excisional depth are particularly important. The power setting of 2 or 3 allowed an efficient and safe debridement in this case. Sharp techniques do not work well in small areas and in areas with a three-dimensional structure and the Versajet™ demonstrated particular advantage in the debridement of superficial to mid-thickness burns in areas like the face, hand, and foot which can be difficult to reach and contour with conventional techniques [2,3].

**Figure 17:**
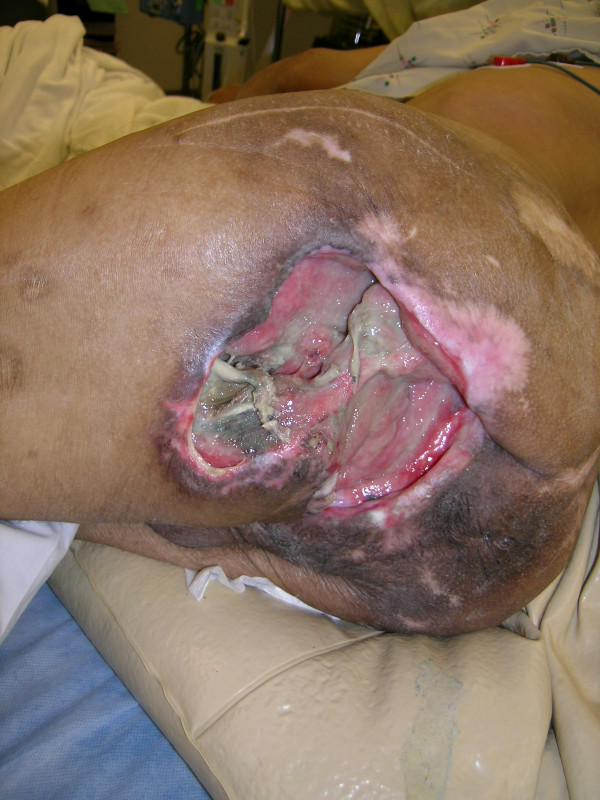
A complex contour pressure sore involving sacral, gluteal and ischial regions in a 48-year-old paraplegic patient.

**Figure 18:**
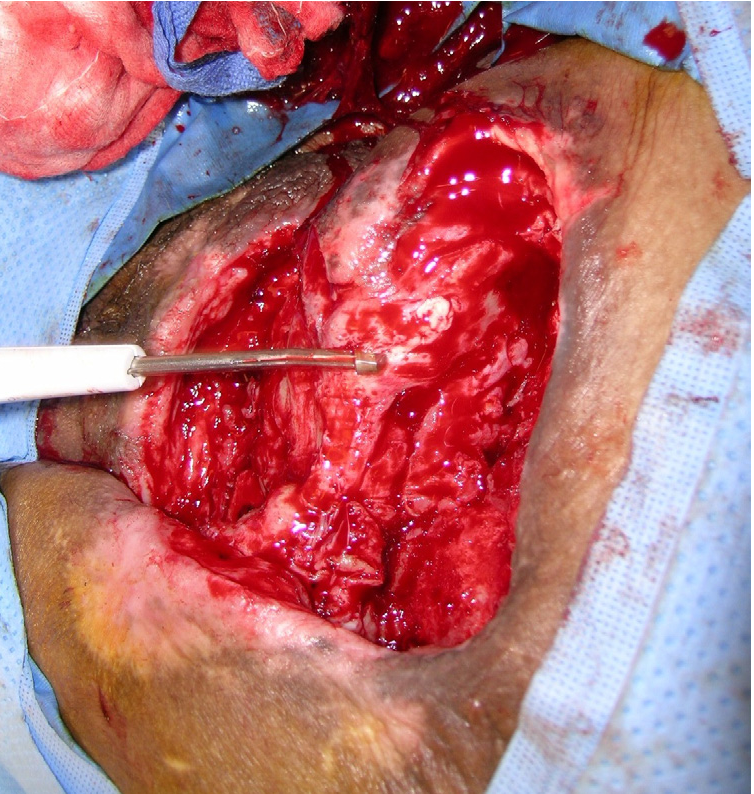
Use of Versajet™ for debridement of this wound with irregular surfaces before placement of wound V.A.C.

Another significant advantage of this device demonstrated was its ability to efficiently debride irregular and complex contour wounds such as deep pressure sores, traumatic wounds which can often be difficult to effect with cold knife (Patient 6, Fig. 17, 18). However, because in its current form, the device does not effectively debride desiccated eschar in pressure sores covered with dry eschar, the preferred approach is to sharply remove the eschar and then use the Versajet™ to debride the underlying necrotic tissue.

The system enables rapid and controlled debridement, likely resulting in shorter procedure times. In our experience it reduced total operating room time for debridement with the estimated time of debridement on average approximately 1.5 times higher if Versajet™ debridement was not used. Although the scope of this paper is not to study cost-effectiveness of the system, but to describe our experiences with the Versajet™, there is some evidence indicating significant cost savings and reduction of the number of required debridement procedures with hydrosurgery system compared to conventional techniques. Despite the high cost of the disposable handpiece (355 $), it has been indicated that the hydrosurgical approach shortens hospitalization and healing time, thus allowing a total savings in wound management [1,4]. However, none of these studies^1,4 ^have included a well-designed control group to demonstrate conclusively that the instrument is cost effective. Further studies in this field that include a true control group in their study design are required.

Nevertheless, based on our experience, we believe that the hydrosurgery system worked more efficiently in the presented case series for wound preparation and debridement with a satisfactory outcome compared to conventional sharp techniques utilized in similar control wounds.

We have been using this hydrosurgery system for wound debridement for approximately one year and based on my sixteen-year experience in debridement of a variety of similar wounds with conventional sharp techniques and subsequent reconstruction, a comparison between conventional sharp debridement and hydrosurgery system is provided in the following paragraphs, in addition to pertinent comparison data from few available published reports.

Chronic venous ulcers and diabetic wounds of the lower extremity were debrided more efficiently with the hydrosurgery system compared to the sharp knife. Versajet™ gradually removed the necrotic and/or fibrinous tissue and enabled us more precisely to preserve the viable tissue for a possible skin grafting. In such cases, the outcome was significantly better in Versajet™ group.

Deep pressure sores, traumatic complex deep perineal wounds, traumatic deep extremity wounds with irregular surfaces were often hard to debride with sharp techniques, and we found the handpiece of the Versajet™ very efficient for debridement of the deep necrotic tissues. Similarly, in traumatic degloving type injuries where avulsion skin flaps developed, it was often very difficult to debride necrotic tissues under those flaps using conventional technique. By means of the handpiece, Versajet™ provided faster and more effective debridement that also allowed the flaps to easily attach to the recipient wound bed. Furthermore, sharp knife sometimes caused unexpectedly deep debridements jeopardizing the viability of the avulsed flaps. A clinical comparison revealed a better postoperative outcome in cases where hydrosurgery system was used.

In our experience, in deep second degree as well as third degree burn injuries involving large flat surfaces, tangential excision using sharp knife has always been a superior option. In addition, using the Versajet™ in such cases increased the time required for debridement, in addition to its cost. However, in small three dimensional areas requiring debridement Versajet™ proved to be a better alternative.

There was no difference between Versajet™ and conventional techniques in terms of operating time required for debridement, number of the debridements and outcome when treating superficial second degree burn wounds and superficial traumatic or postsurgical wounds, regardless of wound size. However, Versajet™ proved to be costly when used in such cases.

Versajet™ was not an option for removing eschar. Furthermore Versajet™ was not effectively applicable in wounds where the bulk and amount of necrotic tissue load was very high. In such cases, conventional sharp techniques provided faster and efficient debridement and better outcome. Nevertheless one can remove the bulky necrotic tissue with sharp techniques and use Versajet™ for a precise debridement in such cases. However cost of the treatment would be an issue in such cases.

Use of Versajet™ seemed to decrease the number of the debridements required in preparation of the wounds in our cases. This was evidenced by the decreased postoperative wound infection and better reconstructive outcome with higher skin graft take rate. In decubitus ulcers, venous ulcers, chronic ulceration from traumatic or postsurgical wounds, Granick [4] et al. demonstrated a statistically significant reduction in the number of debridements required to adequately prepare the wound bed for closure in Versajet™ group compared to conventional techniques. This seemed to be associated with more efficient debridement leading to decreased bacterial count. Mosti [1] et al. compared the Versajet™ and traditional moist dressing in leg ulcers and found that bacterial burden decreased from 10 ^6 ^to 10 ^3 ^in approximately 42 % of the patients in the Versajet™ group. However, a randomized controlled study seems necessary to demonstrate the effects of the Versajet™ system on quantitative bacterial load as compared to conventional techniques.

## Conclusion

Efficient debridement of traumatic wounds, pressure sores, burn wounds, and chronic non-healing wounds due to diabetes mellitus, venous insufficiency, peripheral vascular disease is an essential and crucial step in wound management. In this article, usage of a hydrosurgery system that utilizes high fluid technology is presented for debridement of various wounds in fifteen patients. Technical details and pitfalls are provided to facilitate clinical application of the technique. Inability to remove hard eschar and to debride the bone are two known drawbacks of the hydrosurgery system. Even though the hydrosurgery system cannot replace sharp techniques for desiccated eschar removal and other techniques for bone debridement, it can be an efficient alternative for soft tissue debridement in certain cases. We believe that this new tool will soon find greater applicability in our practice. However, its cost effectiveness needs to be studied in detail with well-controlled studies.

## Competing interests

The author has no financial interest and is not a shareholder in Smith & Nephew.
